# Technique Advances in Enteroatmospheric Fistula Isolation After Open Abdomen: A Review and Outlook

**DOI:** 10.3389/fsurg.2020.559443

**Published:** 2021-01-20

**Authors:** Jinjian Huang, Huajian Ren, Yungang Jiang, Xiuwen Wu, Jianan Ren

**Affiliations:** Research Institute of General Surgery, Jinling Hospital, School of Medicine, Southeast University, Nanjing, China

**Keywords:** open abdomen, enteroatmospheric fistula, fistula isolation, biomedical device, 3D printing

## Abstract

Enteroatmospheric fistula (EAF) after open abdomen adds difficulties to the management and increases the morbidity and mortality of patients. As an effective measurement, reconstructing gastrointestinal tract integrity not only reduces digestive juice wasting and wound contamination, but also allows expedient restoration of enteral nutrition and intestinal homeostasis. In this review, we introduce several technologies for the temporary isolation of EAF, including negative pressure wound therapy, fistuloclysis, fistula patch, surgical covered stent, three-dimensional (3D) printing stent, and injection molding stent. The manufacture and implantation procedures of each technique with their pros and cons are described in detail. Moreover, the approach in combination with finger measurement, x-ray imaging, and computerized tomography is used to measure anatomic parameters of fistula and design appropriate 3D printer-recognizable stereolithography files for production of isolation devices. Given the active roles that engineers playing in the technology development, we call on the cooperation between clinicians and engineers and the organization of clinical trials on these techniques.

## Introduction of Open Abdomen Therapy

The open abdomen therapy can be chosen for severe intraabdominal infections and abdominal compartment syndrome (ACS) when no other perceived options exist. This strategy allows surgeons to carry out source control procedures on unexplored abdominal infections and reduce intraabdominal pressure for the prevention of visceral organ ischemia (1). Early closure of abdomen is highly recommended once patients' conditions are improved because open abdomen therapy can alter the normal physiological states of abdomen and cause wound infections, seromas, fistula formation, recurrence of the defect, and even death (2–4). Primary fascia closure is an ideal solution to realize the abdominal closure, but in the presence of large fascia defects, temporary abdominal closure (TAC) can be alternatively applied including Bogotà bag, skin closure, Wittmann patch, Barker vacuum pack, commercial negative pressure wound therapy (NPWT), and commercial NPWT plus mesh-mediated traction (5). A clinical investigation from the International Register of Open Abdomen (IROA) study group indicated that NPWT was the most frequent choice (46.8%) for TAC (6, 7) because it facilitated the formation of wound granulation, prevention of fistula formation, and reduction of wound contaminations (8, 9). Even with these interventions, the occurrence of enteroatmospheric fistula (EAF) still reached 9% and the overall mortality rate in the entire population was 29.7%. In the subgroup analysis, it was revealed that EAF raised the death rate from 28.8 to 39%, suggesting that EAF was an independent risk factor of mortality as it can lead to the loss of digestive fluid and other complications including wound contamination, water-electrolyte disturbance, troubles in enteral nutrition, and hyper-metabolic conditions as well as chronic intestinal failure (10, 11). Moreover, once the mucosa is protruded, EAF cannot be closed spontaneously and has to be resected until the patient has recovered and the wound completely heals (12). For this reason, fistula isolation is very important to ensure the safety of patents waiting for definite fistula surgeries.

## Emerging Techniques for EAF Isolation

EAF requires comprehensive treatments: (1) nutritional support, among which the early total parenteral nutrition is beneficial for intestinal rest and spontaneous fistula closure; (2) somatostatin analogs, which reduce gastrointestinal (GI) secretions and allow fast fistula closure, but do not reduce the mortality. Most of the studies agree that the greatest benefit occurs in the first 10 days of treatment (13, 14); (3) antibiotics, whose application should follow the Surviving Sepsis guidelines, and empiric coverage should not exceed 4 to 7 days (15, 16); (4) maintenance of water and electrolyte balance. Fluid infusion is administered based on a general analysis of fistula's output and body fluid balance; (5) others, such as fibrin glue, endoscopic clips, and fistula plug can be considered the adjuvant therapy for non-operative fistula closure (17–20). In addition, various EAF isolation approaches have been invented for improving the wound protection and maintaining the GI homeostasis, therefore playing increasing therapeutic roles.

### NPWT

**Figure 1:**
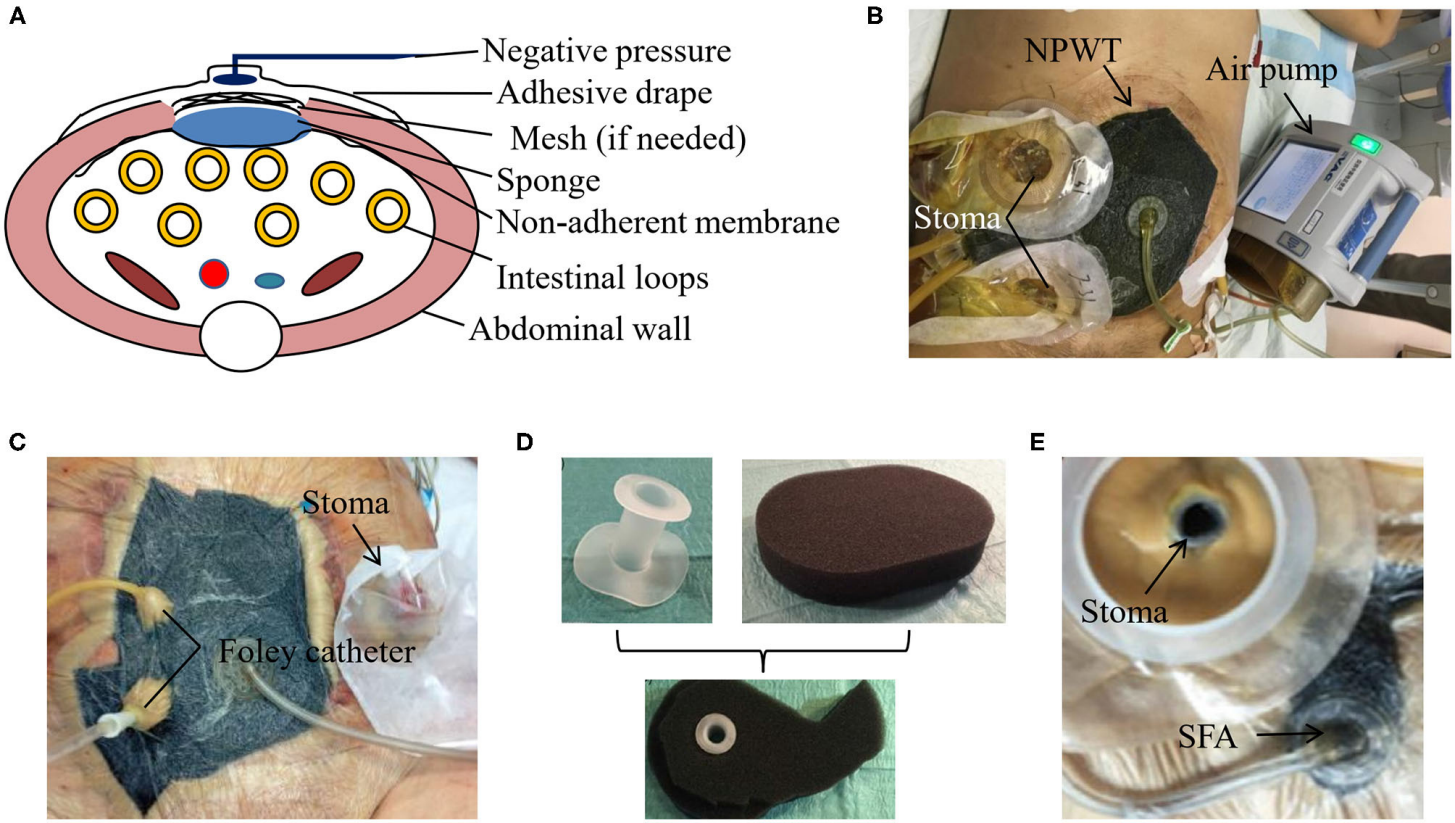
NPWT for EAF isolation. **(A)** The schematic diagram of the vacuum-assisted closure (VAC) device for NPWT. **(B)** The patient with EAF received NPWT in our medical center. The use of picture was approved by the patient. **(C)** Foley catheter can enhanced the drainage of EAF with protruded mucosa. Reproduced with the permission from Bobkiewicz et al. **(D,E)** The real product of the silicone fistula adapter (SFA) and its application in EAF for an efficient drainage. Reproduced with the permission from Wirth et al.

NPWT has been widely used for TAC in clinical practice. Firstly, the skin necrosis and any other necrotic tissues need to be debrided. Secondly, an obligatory non-adherent polyvinyl alcohol membrane serves as the first layer of NPWT over the intestinal loops and a piece of white sponge as the second. The non-adherent layer can effectively prevent the adhesion between the intestinal loops and the sponge. It is worth noting that the sponge needs to be tailored slightly smaller than the size of abdominal wound to leave enough space for abdominal wall traction. If there are any skin folds, stoma and drainage tubes, the stoma pastes or silicone gels are required to make the entire negative pressure system sealed. Finally, the adhesive drape is placed over the sponge with the margins of intact skin. An external negative pressure is chosen ranging from −100 to −125 mmHg to drain the intestinal fluids according to their output, the number of EAFs, the amount of NPWT, and the process of wound healing (21). The whole equipment for NPWT is described in Figure 1A, which has been commercially available from KCI (TX, USA). Figure 1B shows the practice of NPWT in our medical center.

Although treated with NPWT, the spontaneous closure of EAF is very rare and depends on the fistula location and output (22, 23). Once the mucosal protrusion of EAF occurs, spontaneous closure becomes impossible, and the only chance for patients is to survive with the intention of further surgery. In this situation, further aggressive approaches can be considered if a satisfied source control has not been achieved. For example, Foley catheter can be placed directly into the intestine lumen and pulled out through holes in every layer of the NPWT dressing (Figure 1C), which enables drainage of high-output fistula and achieves a more satisfying result than the NPWT alone (21). Similarly, the silicone fistula adapter (SFA) has been invented by PPM Fistelapater to realize the isolation of fistula opening in combination with NPWT (Figures 1D,E) and drains the EAF more efficiently (24).

### Fistuloclysis

**Figure 2:**
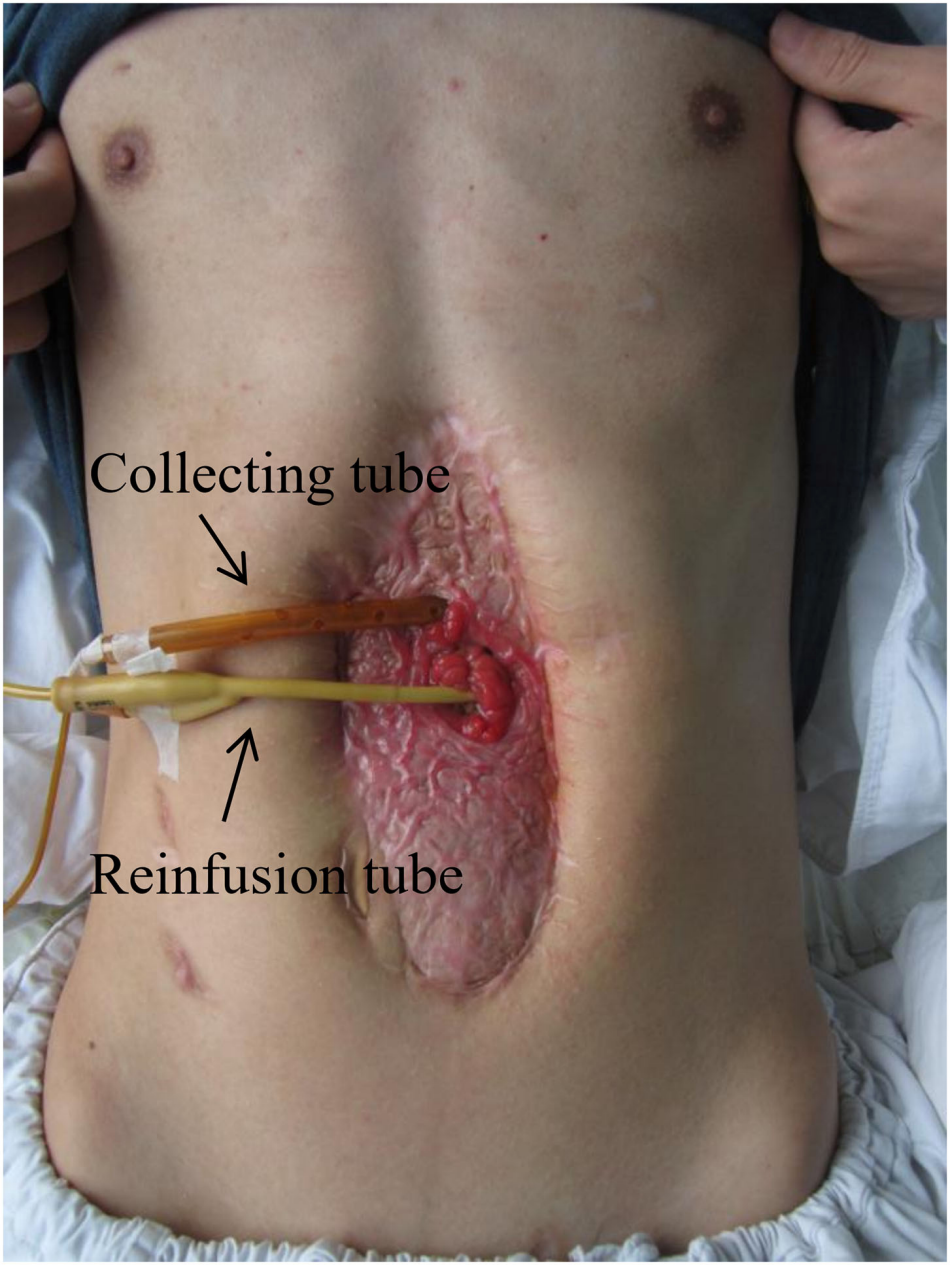
Fistuloclysis for the restoration of enteral nutrition. This patient was treated with open abdomen (grade 4, frozen abdomen) and the effluent was immediately reinfused into distal limb of EAF. The use of picture was approved by the patient.

Management of EAF requires sufficient nutritional and metabolic supports by the means of gradual transition from parenteral nutrition to enteral nutrition. Long-term parenteral nutrition may lead to complications such as the catheter-related bloodstream infections, liver injury, and intestinal dysfunction, while enteral nutrition can improve those conditions. As an enteral nutrition routine, fistuloclysis can be used to infuse enteral nutrients, formula, or proximal GI secretions to the distal limb of fistula in order to improve the nutritional status and maintain fluid/electrolyte homeostasis (25, 26). Figure 2 shows that this technique was applicable in granulating the open abdomen with EAF in our medical center. It was revealed by Mettu et al. (27) that fistuloclysis could replace the parenteral nutrition and reduce the cost of nutritional support. For these critically ill surgical patients, fistulaclysis allows early enteral nutrition, which improves prognosis through improvements of intestinal barrier functions and immune states (28, 29).

However, fistuloclysis has been less popular in recent years mainly due to technical and aesthetic concerns (30). Some EAFs are not appropriate for fistuloclysis because of their distal locations or failure in the placement of feeding tubes. In addition, this approach is accomplished with risks of the tube dislocation, effluent deterioration, and wound contamination, thus consuming large amounts of nursing work. Through combining with NPWT, it facilitates the control of fistula effluent and fixation of fistuloclysis tube, which makes fistuloclysis safer (31). Collectively, our opinion is to carry out this technique if enteral nutrition is achieved especially when EAF is located in the proximal part of small intestine, and this approach will enhance the physical strength of patients to tolerate definite surgery.

### Fistula Patch

**Figure 3:**
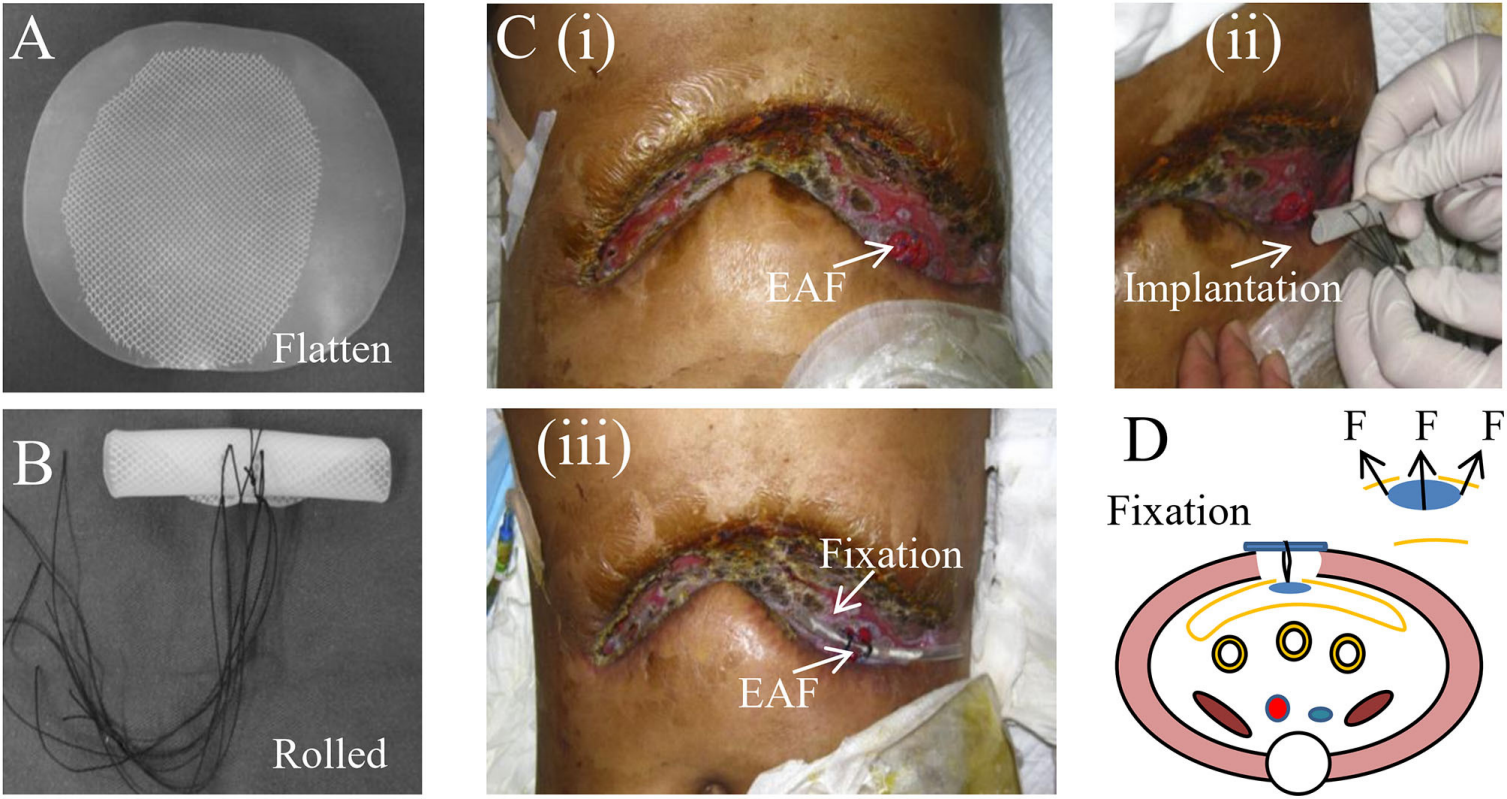
Fistula patch for the occulsion of EAF. **(A)** The appearance of fistula patch. **(B)** The fistula patch needs to be rolled up for implantation. **(C)** The patients with EAF received the treatment of fistula patch that was fixed using a tube. Reproduced with the permission from Wang et al. **(D)** The schematic diagram of the fistula patch application and fixation. It highlights the potential risks for cutting the intestinal wall caused by the patch edge.

Compared with fistuloclysis, intraluminal occlusion of EAF is more advantageous since it supports the physical integrity of GI tract and ensures the pass of digestive contents with less leakage from EAF. Fistula patch is the first-generation device that addresses this issue. As shown in Figure 3A, the patch is made of two pieces of silica gels embedded with a polypropylene mesh, combining the materials' elasticity and plasticity. This design enables the rolling of the patch for implantation as well as rapid shape recovery in the intestinal lumen (Figure 3B).

Notably, the patch can only be applied to the EAFs with mucosal protrusion and needs to be tailored in accordance with their anatomic characteristics. Figure 3C shows a patient treated with the fistula patch, which was fixed above the abdominal wounds using a tube. A study from our medical center revealed that this technique could help restore enteral nutrition and reduce the effluent of EAF (32). However, only around half of patients were suitable for this treatment because of the implanting difficulties and anatomic complexity of EAFs. Moreover, the safety concerns regarding this technique have also been raised particularly in these patients with intestinal edema or anatomic mismatch of the patch to the EAF. This is because under those conditions, the intestinal wall is prone to the physical cutting of the patch edge (Figure 3D), leading to GI rupture and bleeding, although these complications are rare.

### Fistula Stent

**Figure 4:**
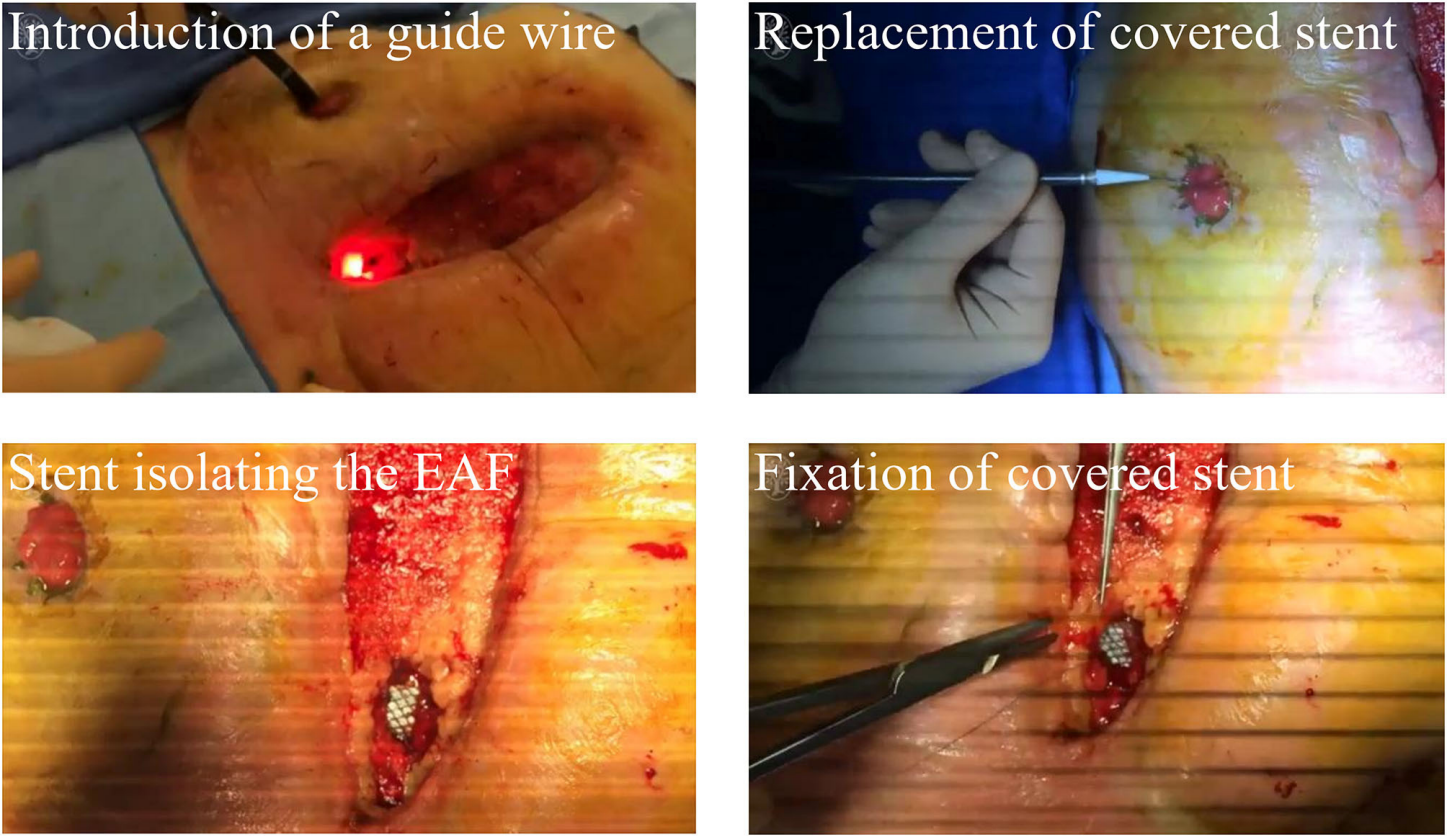
A commercial covered self-expanding metal stent was used to isolate the EAF. Reproduced with the permission from Rebibo et al.

More insights have been put on the fistula stent that can be made tubularly and conforms to the shape of intestinal tract. As shown in Figure 4, Rebibo et al. (33) reported a covered self-expanding metal stent (Hanarostent HRC, Life Partners Europe, Bagnolet, France) to close the EAF. This stent was implanted through a terminal ileostomy assisted by a guide wire and the endoscopy. Three patients received this treatment and regained the enteral nutrition. Combined with NPWT, two of them achieved the spontaneous closure of EAF, and then the stent could be removed in the assistance of endoscopy. This study preliminarily indicated the effectiveness of the covered self-expanding metal stent. However, pre-existing ileostomy and complicated implanting process greatly limit the indication of this management for EAF.

**Figure 5:**
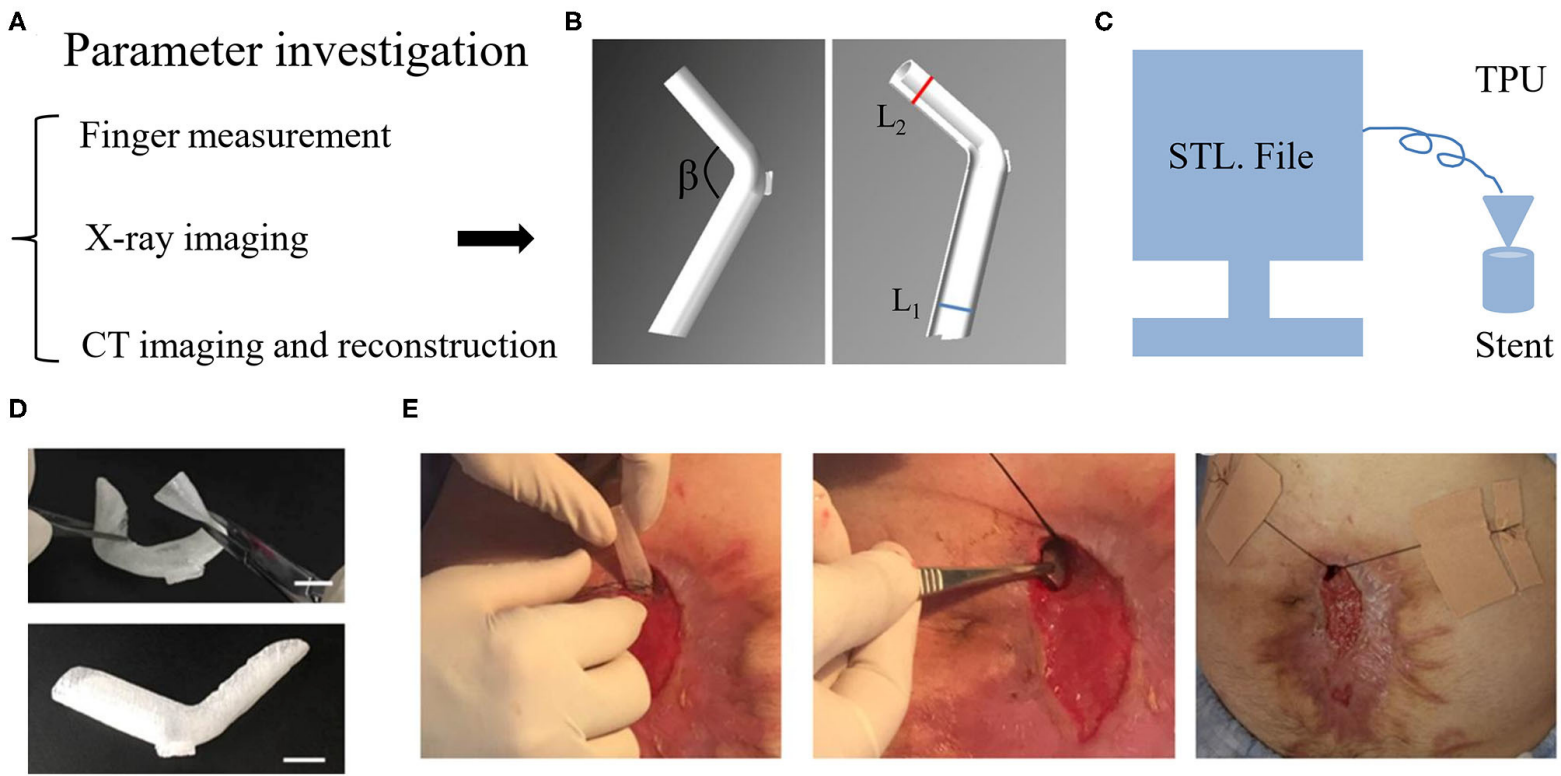
3D printing stent for the isolation of EAF. **(A)** The methods to measure the anatomic parameters of EAF. **(B)** The STL file of stent designed using Solidwork software according to the measurement of EAF anatomy. **(C)** The schematic diagram of a 3D printer run in the FDM model to print the stent. **(D)** Implantability of the stent due to its shape flexibility. **(E)** The process of the stent implantation and fixation. Reproduced with the permission from Huang et al.

Our medical center explored a novel approach that extended the application of fistula stent to patients without ileostomy by using a 3D printing technique (34–37). This technique was based on a fused deposition model (FDM) in which the thermoplastic polyurethane (TPU) filament was melted and reshaped as desired. To obtain an appropriate stent, the first step was to investigate the anatomic parameters of EAF. Three methods can be used to measure the EAF including the finger palpation, x-ray imaging, and computerized tomography (CT) (Figure 5A). The finger palpation is the most direct way. By wearing sterile gloves, fingers can reach into the intestinal lumen though the EAF orifice; however, this does not always work when the orifice is too small or the intestinal lumen is too narrow. Moreover, due to the inaccurate and limited touch, some tiny fistulas are easily omitted. Fistulography by the x-ray imaging can be used to detect these tiny fistulas and achieve a general view toward the GI tract integrity (38). Contrast-mediated high resolution CT is a more advantageous method not only due to its high sensitivity for the fistula (39), but also because the images can be reconstructed in 3D so that we are able to visualize the anatomic parameters of EAF (34). The goal of these detection approaches is to obtain the diameters of proximal and distal limbs (L_1_, L_2_) and their resulting angle (β) in order to design a suitable stereolithography (STL) file based on the measurements (Figure 5B). This STL file can be recognized by an FDM 3D printer to print a fistula stent made of TPU (Figure 5C). Because of the elasticity of TPU, the resulting stent can be remodeled during the implantation (Figure 5D). Figure 5E demonstrates a case who received a 3D printing fistula stent for isolation of the EAF. This treatment allowed the patient to restore the enteral nutrition, reduce the effluent, and feel free when doing some exercise for the recovery of physical strength. Notably, the anatomic features of EAF may be altered with the change of intestinal adhesion; therefore, a new stent is needed at this situation to replace the old, particularly in case the effluent greatly increased. Considering the implanting difficulties, the stent is only indicated to a relatively large orifice of EAF with mucosal protrusion. Moreover, the complicated manufacturing process limited the promotion of this technology to other medical centers so that the current clinical evidence is low.

**Figure 6:**
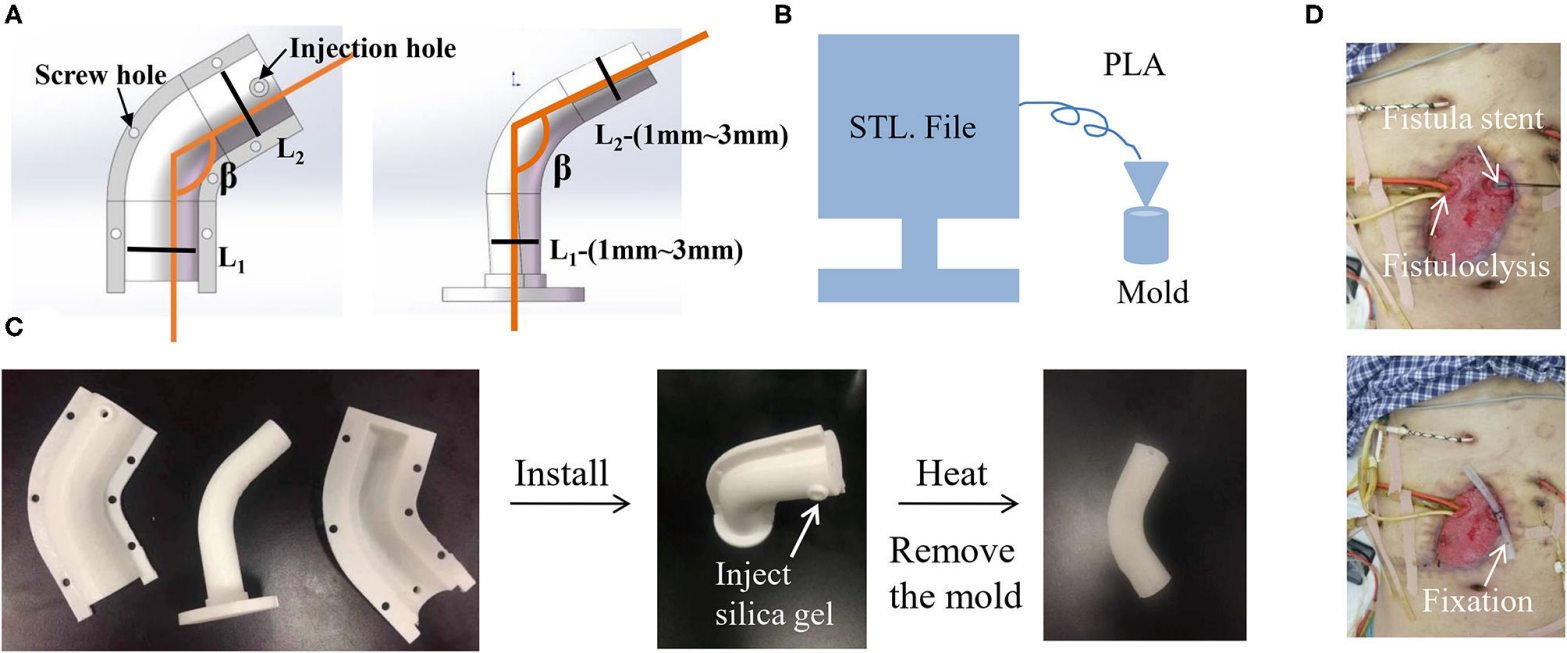
Injection molding stent as a universal approach for the isolation of EAF. **(A)** The STL file of mold designed by Solidwork software according the measurement of EAF anatomy. **(B)** The schematic diagram of an FDM 3D printer printing the mold. **(C)** The real product of the printed mold and the stent that was casted from the mold. **(D)** The application of an injection molding stent in combination with fistuloclysis for the treatment of multiple EAFs. The use of picture was approved by the patient.

As an alternative, injection molding stent was invented and was easily promoted because the molds can be prepared in advance based on different anatomic parameters of EAF with the β ranging from 70° to 180° and the parameters ranging from 1 cm to 3 cm. The extreme case still requires a 3D printing stent or to customize a mold. As shown in Figure 6A, the mold consists of two shells and one core. The shell is tailored with the holes at the edge for fixation and the hole in the middle for injection of silica gel, which fills up the space between the shell and core and then is solidified at 100°C for 30 min. The mold is fabricated using a 3D printer in FDM and made of the polylactic acid (PLA) (Figure 6B). Figure 6C demonstrates the manufacturing process of a stent casted by a mold from the mold installation, silica gel injection, to the mold removal. The silica gel product is featured on its elasticity so that the stent is easily implanted into EAF (Figure 6D). This technique consumes less time (merely 1h) to access a personalized stent compared with the 3D printing stent. Because of a similar size to the 3D printing stent, this injection molding stent is also only indicated to a relatively large orifice of EAF with mucosal protrusion. Table 1 lists the comparisons among all mentioned approaches from technical and clinical perspectives, and the choice is based on the individual conditions of patients.

**Table 1:** Comparisons of fistula isolation approaches.

	**NPWT**	**Fistuloclysis**	**Fistula patch**	**Covered metal stent**	**3D printing stent**	**Injection molding stent**
Commercially available	Yes	Yes	Yes	Yes	No	No
Average time for device preparation and implantation (hour)	0.5	0.2	0.2	Not known	12-24	1
Contraindications	Not specific	Fistula distally located; distal tract not accessed.	Small fistula orifice	Without intestinal stoma	Small fistula orifice	Small fistula orifice
Potential technical risks	Not specific	Chyme contamination	Mechanical damage to adherent mucosa	Iatrogenic injury (endoscopic perforation)	Iatrogenic injury during implantation	Iatrogenic injury during implantation
Clinical evidence	High	Moderate	Low	Low	Low	Low

## Outlook for EAF Management

Although the progress has been achieved on EAF isolation, the technique still needs to be further optimized. First, due to the limited contrast between intestinal tissues and the surrounding soft tissues, current imaging methods cannot extract the intestinal photos directly so that we have to design the STL file separately using the Solidwork software, which is time-consuming. Magnetic resonance imaging (MRI) technology has shown higher sensitivity in distinguishing GI fistula from soft tissues (40, 41), and is promising to provide a more precise tool for the measurement of EAF anatomy. When combined with artificial intelligence, the ultimate goal is to achieve the STL file of fistula stents directly from the fistula images in a quick and convenient manner (42–45). Moreover, the small size of EAF's orifice hinders the implantation of the fistula stent. To address this issue, elastic and shape-memory biomaterials should be tested for production of the stent (46–48). In addition, the clinical trials based on these techniques are urgently needed for providing more evidence.

## Conclusion

In this review, we introduced several isolation approaches for the management of EAF after open abdomen including NPWT, fistuloclysis, fistula patch, surgical covered stent, 3D printing stent, and injection molding stent. The choice of these approaches should consider the condition of EAF, general body habitus, and the treatment purpose. The fistula stent is a new solution with promising functions in the maintenance of the GI tract physical integrity. The cooperation between surgeons and engineers is advocated to promote the improvement and application of these techniques.

## Author Contributions

JR and XW conceived this review. JH organized literatures and wrote this review. HR organized the photographs and the table. YJ participated in the discussion. All authors contributed to the article and approved the submitted version.

## Conflict of Interest

The authors declare that the research was conducted in the absence of any commercial or financial relationships that could be construed as a potential conflict of interest.

## Abbreviations

EAF: enteroatmospheric fistula
NPWT: negative pressure wound therapy
CT: computerized tomography
STL file: stereolithography file
ACS: abdominal compartment syndrome
TAC: temporary abdominal closure
GI: gastrointestinal
FDM: fused deposition model
PLA: polylactic acid
TPU: thermoplastic polyurethane
MRI: magnetic resonance imaging.
